# Functional Characterization of a New Degradation Peptide BmTX4-P1 from Traditional Chinese Scorpion Medicinal Material

**DOI:** 10.3390/toxins15050340

**Published:** 2023-05-15

**Authors:** Chenhu Qin, Xuhua Yang, Yuanyuan Zhang, Gang Deng, Xin Huang, Zheng Zuo, Fang Sun, Zhijian Cao, Zongyun Chen, Yingliang Wu

**Affiliations:** 1 College of Life Sciences, Wuhan University, Wuhan 430072, China; 2016202040025@whu.edu.cn (C.Q.); yangxh@whu.edu.cn (X.Y.); 2021202040010@whu.edu.cn (Y.Z.); 2020202040145@whu.edu.cn (G.D.); 2013301060077@whu.edu.cn (X.H.); 2014301060068@whu.edu.cn (Z.Z.); 2016202040055@whu.edu.cn (F.S.); zjcao@whu.edu.cn (Z.C.); 2 Department of Biochemistry and Molecular Biology, College of Basic Medicine, Hubei University of Medicine, Shiyan 442000, China; 3 Center for BioDrug Research, Wuhan University, Wuhan 430072, China

**Keywords:** thermally processed scorpions, BmTX4-P1, chemical synthesis and recombinant expression, electrophysiology experiments, hKv1.2 and hKv1.3 channels

## Abstract

Thermally processed *Buthus martensii* Karsch scorpion is an important traditional Chinese medical material that has been widely used to treat various diseases in China for over one thousand years. Our recent work showed that thermally processed *Buthus martensii* Karsch scorpions contain many degraded peptides; however, the pharmacological activities of these peptides remain to be studied. Here, a new degraded peptide, BmTX4-P1, was identified from processed *Buthus martensii* Karsch scorpions. Compared with the venom-derived wild-type toxin peptide BmTX4, BmTX4-P1 missed some amino acids at the N-terminal and C-terminal regions, while containing six conserved cysteine residues, which could be used to form disulfide bond-stabilized α-helical and β-sheet motifs. Two methods (chemical synthesis and recombinant expression) were used to obtain the BmTX4-P1 peptide, named sBmTX4-P1 and rBmTX4-P1. Electrophysiological experimental results showed that sBmTX4-P1 and rBmTX4-P1 exhibited similar activities to inhibit the currents of hKv1.2 and hKv1.3 channels. In addition, the experimental electrophysiological results of recombinant mutant peptides of BmTX4-P1 indicated that the two residues of BmTX4-P1 (Lys^22^ and Tyr^31^) were the key residues for its potassium channel inhibitory activity. In addition to identifying a new degraded peptide, BmTX4-P1, from traditional Chinese scorpion medicinal material with high inhibitory activities against the hKv1.2 and hKv1.3 channels, this study also provided a useful method to obtain the detailed degraded peptides from processed *Buthus martensii* Karsch scorpions. Thus, the study laid a solid foundation for further research on the medicinal function of these degraded peptides.

## 1. Introduction

Traditional Chinese medicinal materials refer to the collection of parts or all of plants or animals in nature that are processed to obtain materials for treating diseases, guided by traditional Chinese medical theory, and have been used to treat diseases for a very long history in China. Thermally processed *Buthus martensii* Karsch scorpion, as a routine animal medicinal material in traditional Chinese medicine, has been used for over a thousand years to treat various diseases, including epilepsy, rheumatoid arthritis, and chronic pain [1]. The scorpion medicinal material is usually ground into powder and taken orally by patients after mixing it with other medicinal materials [1]. Based on the Chinese saying “combat poison with poison”, the venom gland from the medicinal material is an effective medicinal part. However, to date, the effective medicinal components from the venom gland of this medicinal material and their mechanism of action are not well understood.

The traditional Chinese scorpion medicinal materials are obtained from wild *Buthus martensii* Karsch scorpions. To date, there have been many studies on neurotoxins from the venom of *Buthus martensii* Karsch scorpions, from which a large number of neurotoxins that act on ion channels have been found [2,3,4,5,6,7]; some neurotoxins were considered to have the potential to treat diseases [8,9,10,11,12]. However, there had been no report on the peptide components of scorpion medicinal materials for a long time. Recently, our group performed a peptidomics study on the venom glands of scorpion medicinal materials [13]. A large number of degraded peptides are still present in the venom gland of scorpion medicinal materials [13]. Therefore, investigating the pharmacological properties and the structure–function relationship of these degraded peptides identified in traditional Chinese scorpion medicinal materials is a new challenge.

In this study, we identified a new degraded peptide, BmTX4-P1, from traditional Chinese scorpion medicinal materials. The BmTX4-P1 peptide degraded some amino acids at the N-terminal and C-terminal regions compared with the venom-derived peptide toxin BmTX4. The BmTX4-P1 peptide was prepared by two methods (i.e., chemical synthesis and recombinant expression). The synthesized peptide sBmTX4-P1 exhibited high activity to inhibit the currents of hKv1.2 and hKv1.3 channels, with half-inhibitory concentrations of 74.7 ± 7.0 nM and 123.9 ± 26.7 nM, respectively. The recombinant peptide rBmTX4-P1 showed similar activities to inhibit currents of hKv1.2 and hKv1.3 channels, with IC_50_ values of 45.7 ± 9.2 nM and 246.8 ± 47.9 nM, respectively. Many papers have reported that potassium channels hKv1.2 and hKv1.3 are closely related to various diseases, including epilepsy and rheumatoid arthritis [14,15,16,17,18,19,20,21], which likely coincide with the diseases that this medicinal material can treat. These results indicated that the degraded peptides in traditional Chinese scorpion medicinal materials may play an important role in the treatment of diseases.

## 2. Results

### 2.1. Identification of a New Degraded Peptide, BmTX4-P1, from Traditional Chinese Scorpion Medicinal Material

**Figure 1:**
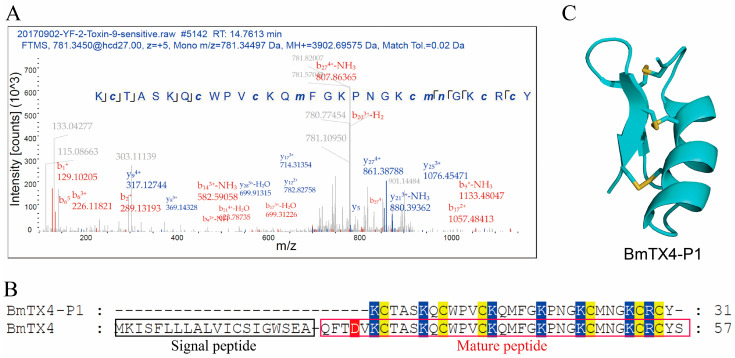
A new degraded peptide, BmTX4-P1, was identified from scorpion medicine materials. (**A**) BmTX4-P1 was identified by MS/MS spectra analysis. Through analyzing the b-ions and y-ions and their derivatives in the spectra, the amino acid sequence of BmTX4-P1 was confirmed. The b-ions and y-ions are shown in red and blue, respectively. The amino acid sequence and sequence coverage of BmTX4-P1 are listed together. (**B**) Sequence alignment of BmTX4-P1 and wild-type toxin peptide BmTX4. The background colors of cysteines, acidic residues, and basic residues are yellow, red, and blue, respectively. (**C**) The 3D structure of BmTX4-P1. Based on the BmTX1 template (PDB code: 6AVC), the 3D structure of BmTX4-P1 was predicted through the SWISS-MODEL server.

Traditional Chinese scorpion medicine material has been used for more than a thousand years to treat various diseases. It has been reported that there are a large number of degraded peptides in the thermally processed scorpion venom gland. However, research on these degraded and thermostable peptides remains in its infancy. Here, we further identified a new degraded peptide, BmTX4-P1, through proteomic analysis according to a previously reported process (Figure 1A) [13]. The b-ions and y-ions of the BmTX4-P1 peptide detected in the spectra revealed the presence of the BmTX4-P1 peptide (Figure 1A). The sequence of BmTX4-P1 showed that BmTX4-P1 contained six conserved cysteine residues (Figure 1B). SWISS-MODEL was applied to predict the 3D structure of BmTX4-P1, the result of which revealed that BmTX4-P1 could form a stable motif with a cysteine-stabilized α-helical and β-sheet (CSαβ) fold (Figure 1C).

### 2.2. Functional Characterization of the Chemically Synthesized Peptide BmTX4-P1 (sBmTX4-P1)

BmTX4-P1 was identified from the venom gland of traditional Chinese scorpion medicinal material. Considering the effective medicinal value of the thermally processed scorpion venom gland, we further investigated the pharmacological properties of the BmTX4-P1 peptide.

**Figure 2:**
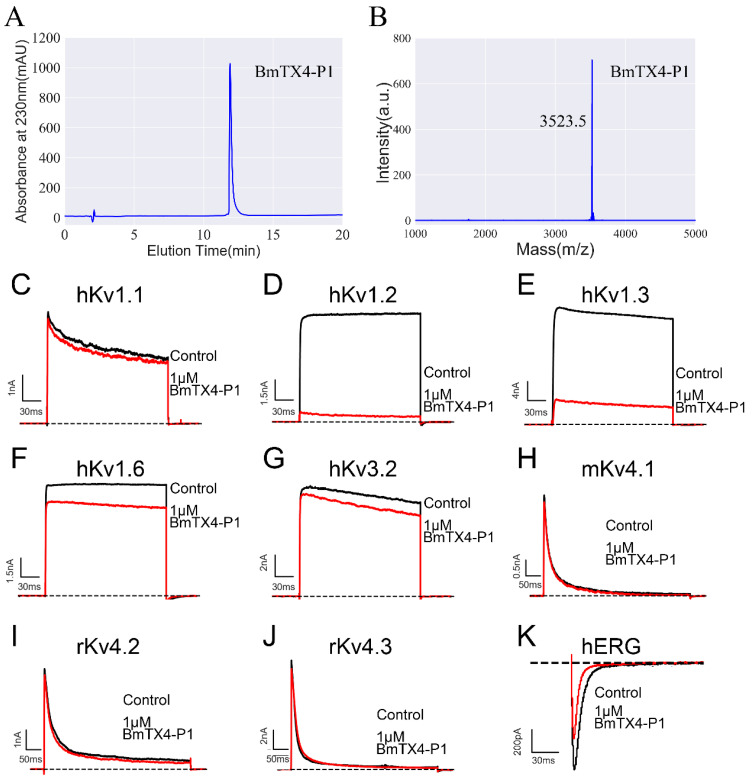
Chemical synthesis of sBmTX4-P1 and its inhibitory effects on potassium channels. (**A**) The purity of synthetic BmTX4-P1 was tested by chromatography. (**B**) Mass spectrometry analysis of sBmTX4-P1. (**C**–**K**) Blocking effects of 1 µM BmTX4-P1 on the currents of different channels. Each channel has been independently tested at least three times, and the inhibitory effects are shown as the mean ± SE.

**Figure 3:**
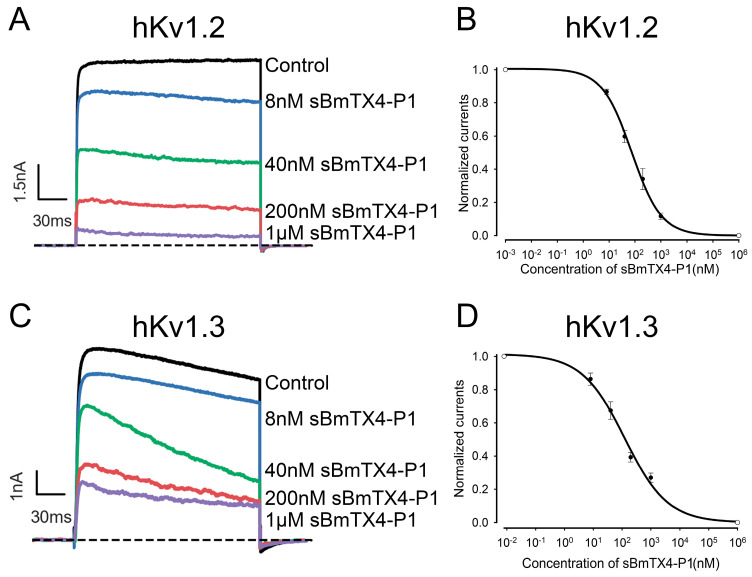
Concentration-dependent inhibition of hKv1.2 channel and hKv1.3 channel currents by sBmTX4-P1. (**A**) The inhibition rates of hKv1.2 channel current by 8 nM, 40 nM, 200 nM, and 1 µM sBmTX4-P1 were 13.5 ± 1.6%, 40.2 ± 3.7%, 66.0 ± 6.3%, and 88.4 ± 2.0%, respectively. (**B**) The average normalized current of hKv1.2 channel was suppressed by sBmTX4-P1 at different concentrations. The IC_50_ value obtained by fitting Hill’s equation is 74.7 ± 7.0 nM. (**C**) The inhibition rates of hKv1.3 channel current by 8 nM, 40 nM, 200 nM, and 1 µM sBmTX were 13.6 ± 3.7%, 32.6 ± 5.3%, 60.7 ± 2.9%, and 73.1 ± 2.8%, respectively. (**D**) The average normalized current of the hKv1.3 channel was suppressed by sBmTX4-P1 at different concentrations. The IC_50_ value obtained by fitting Hill’s equation is 123.9 ± 26.7 nM. The current amplitude in the absence of sBmTX4-P1 was fixed at 1 for the normalized currents, and the inhibition rates of sBmTX4-P1 were compared; the R^2^ values were R^2^_hKv1.2_ = 0.998, and R^2^_hKv1.3_ = 0.995, respectively. Each channel has been independently tested at least three times, and the inhibitory effects are shown as the mean ± SE.

We first prepared peptide sBmTX4-P1 by chemical synthesis. Synthetic BmTX4-P1 was tested for purity by chromatography and then confirmed by mass spectrometry (Figure 2A,B). The measured molecular weight of sBmTX4-P1 is consistent with its calculated value of 3523.3 Da, indicating that we successfully obtained the peptide sBmTX4-P1 (Figure 2A,B). Then, potassium channels (i.e., hKv1.1, hKv1.2, hKv1.3, hKv1.6, hKv3.2, hKv4.1, hKv4.2, hKv4.3, and hERG) were successfully overexpressed, and functional ion channels were assembled in the cell membrane of transiently transfected HEK293 cells. The whole-cell patch clamp technique was applied to test the effects of 1 μM sBmTX4-P1 on these potassium channels. The results showed that 1 µM sBmTX4-P1 inhibited 5.9 ± 1.5% of hKv1.1 channel currents, 88.4 ± 2.0 of hKv1.2 channel currents, 73.1 ± 2.8% of hKv1.3 channel currents, 18.3 ± 4.3% of hKv1.6 channel currents, 5.3 ± 3.1% of hKv3.2 channel currents, 7.0 ± 1.8% of rKv4.1 channel currents, 6.9 ± 4.6% of rKv4.2 channel currents, 6.8 ± 2.8% of mKv4.3 channel currents, and 35.7 ± 2.1 of hERG channel currents (Figure 2C–K). Considering the desirable inhibitory effects of hKv1.2 and hKv1.3 channels by sBmTX4-P1, the IC_50_ values were further deduced from the dose-dependent relationship (Figure 3). sBmTX4-P1 was found to inhibit hKv1.2 and hKv1.3 channels with IC_50_ values of 74.7 ± 7.0 nM and 123.9 ± 26.7 nM, respectively (Figure 3B,D).

### 2.3. Functional Expression and Pharmacological Property Confirmation of Recombinant BmTX4-P1 (rBmTX4-P1)

**Figure 4:**
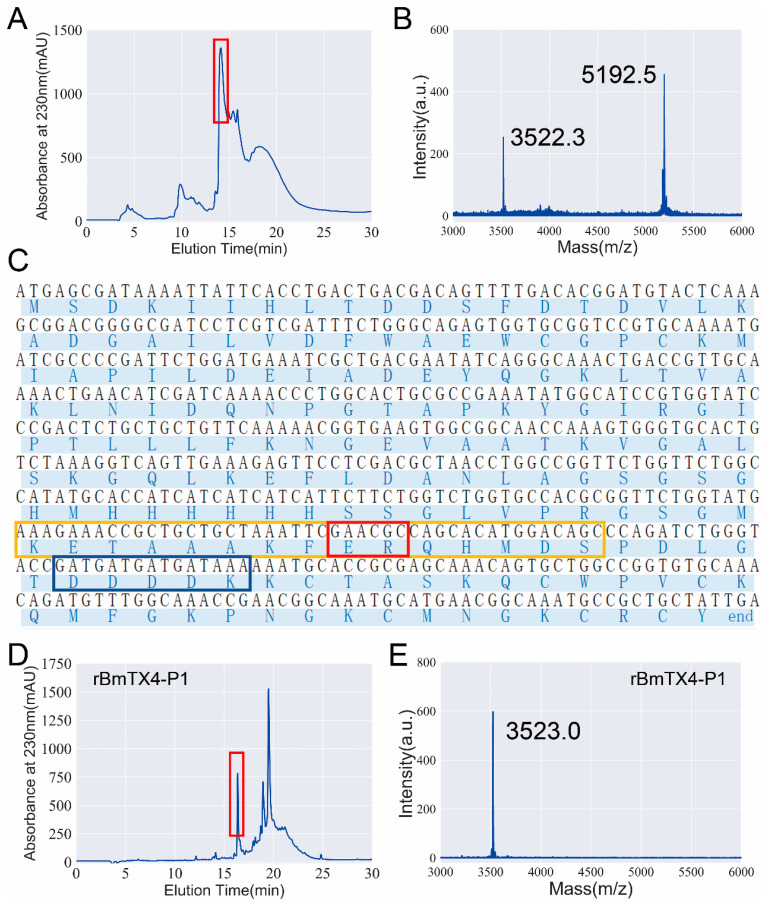
Recombinant expression of BmTX4-P1 (rBmTX4-P1) with a modified pET-32a vector that removed a possible secondary cleavage site of enterokinase. (**A**) The expression products were purified by high-performance liquid chromatography. The products in the red box were collected manually. (**B**) The molecular weight of the product collected in Panel A was identified by mass spectrometry analysis. (**C**) The base sequence and amino acid sequence of the fusion protein. The blue box and the red box indicate the correct recognition site and the wrong recognition site of enterokinase, respectively. The yellow box indicates the region of the 15 aa S-Tag in the expression vector. (**D**) The rBmTX4-P1 was purified by high-performance liquid chromatography. The peak containing rBmTX4-P1 in the red box was collected manually. (**E**) The molecular weight of rBmTX4-P1 in Panel D was identified by mass spectrometry analysis.

**Figure 5:**
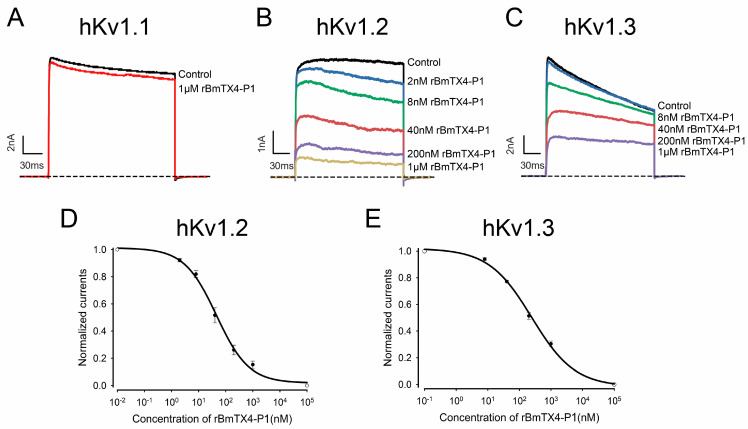
Functional characterization of rBmTX4-P1 on different potassium channels. (**A**) Inhibitory effect of 1 µM rBmTX4-P1 on the current of hKv1.1 channel. (**B**,**C**) Inhibitory effects of different concentrations of rBmTX4-P1 on currents of the hKv1.2 and hKv1.3 channels. (**D**,**E**) The average normalized currents of the hKv1.2 channel and hKv1.3 channel are suppressed by rBmTX4-P1 at different concentrations. The IC_50_ values obtained by fitting Hill’s equation were 45.7 ± 9.2 nM and 246.8 ± 47.9 nM, respectively. The current amplitude in the absence of rBmTX4-P1 was fixed at 1 for the normalized currents, and the inhibition rates of rBmTX4-P1 were compared; the R^2^ values were R^2^_hKv1.2_ = 0.996 and R^2^_hKv1.3_ = 0.998, respectively. Each channel has been independently tested at least three times, and the inhibitory effects are shown as the mean ± SE.

To further investigate the functional residues of BmTX4-P1, recombinant BmTX4-P1 and its mutants were prepared by recombinant expression technique. During the recombinant expression of the BmTX4-P1 peptide, we initially did not obtain the correct BmTX4-P1 peptide after several attempts (Figure 4A,B). The calculated molecular weight of BmTX4-P1 was 3523.3 Da, while there are two measured molecular weights of 3522.3 Da and 5192.5 Da of the expression products (Figure 4B). After confirming that there was no error with the expression vector, enterokinase, and expression steps, we expressed the BmTX4-P1 peptide repeatedly. However, we obtained two measured molecular weights as before. Finally, by analyzing the mass spectrum results and the sequence of the fusion protein (Figure 4B,C), we found that there is a secondary enterokinase cleavage site “ER” in the fusion protein, which is located in the region of the 15 aa S-Tag “KETAAAKFERQHMDS” (Figure 4C). The secondary cleavage site also worked when enterokinase was used to cleave the fusion protein, which resulted in the main recombinant peptide with the wrong molecular weight. The correct and only cleavage site of enterokinase occurs after the amino acid sequence “DDDDK”, but it incorrectly cleaved after the amino acid sequence “ER”, which was consistent with the mass spectrometry results (Figure 4B,C). First, we attempted to replace several other brands of enterokinase to improve its specificity to cleave the fusion protein and expected to obtain the correct rBmTX4-P1, but we obtained the peptide with the wrong molecular weight again. As the fusion protein was purified with a His-tag, an S-tag was not needed in our expression system. To obtain rBmTX4-P1 by recombinant expression in this situation, a new expression vector was constructed by deleting the base sequence “GAACGC” of the expression vector; as a result, the incorrect recognition site “ER” of enterokinase in the fusion protein was deleted (Figure 4C). Finally, the correct recombinant peptide rBmTX4-P1 was obtained with the new expression vector (Figure 4D,E). The correct peptide rBmTX4-P1 was collected manually by high-performance liquid chromatography for 16–17 min and then further analyzed by mass spectrometry (Figure 4D,E). The measured molecular weight of rBmTX4-P1 was 3523.0 Da, which was consistent with its calculated value of 3523.3 Da. Subsequently, rBmTX4-P1 was applied to different potassium channel subtypes through the patch clamp technique to further investigate the pharmacological properties. According to the pharmacological properties of sBmTX4-P1, we investigated the inhibitory effects of rBmTX4-P1 on hKv1.1, hKv1.2, and hKv1.3 channels. The results showed that 1 µM rBmTX4-P1 hardly inhibited the hKv1.1 channel current, while it inhibited the currents of the hKv1.2 and hKv1.3 channels with high affinities, which was consistent with sBmTX4-P1 (Figure 2C–E and Figure 5A–C). The dose–response curve, which was fitted with a Hill equation, showed that rBmTX4-P1 inhibited the hKv1.2 and hKv1.3 channels with IC_50_ values of 45.7 ± 9.2 nM and 246.8 ± 47.9 nM, respectively (Figure 5D,E). These pharmacological properties of rBmTX4-P1 further proved that the functional peptide rBmTX4-P1 was successfully obtained by recombinant expression (Figure 5).

### 2.4. Identification of the Functional Sites of BmTX4-P1 Interacting with the Potassium Channel

**Figure 6:**
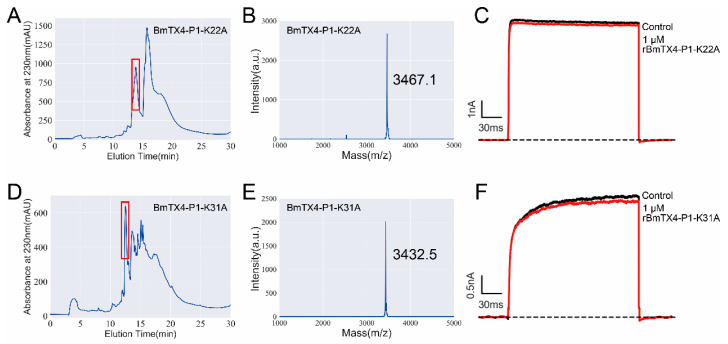

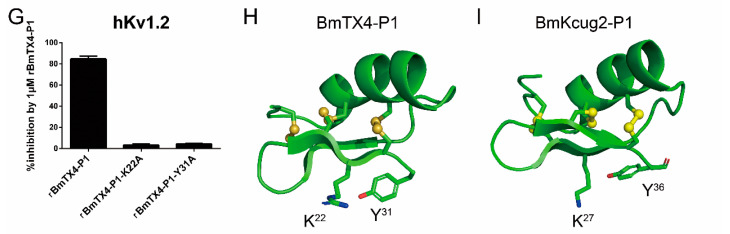
Structure and function relationship of BmTX4-P1 on the Kv1.2 potassium channel. (**A**,**D**) Peak profile of rBmTX4-P1-K22A and rBmTX4-P1-Y31A purified by high-performance liquid chromatography. The peaks containing rBmTX4-P1-K22A and rBmTX4-P1-Y31A are indicated by the red box. (**B**,**E**) Mass spectrometry analysis of rBmTX4-P1-K22A and rBmTX4-P1-Y31A. The measured molecular weights of rBmTX4-P1-K22A and rBmTX4-P1-Y31A are consistent with their calculated values of 3466.2 Da and 3431.2 Da, respectively. (**C**,**F**) Blocking effects of rBmTX4-P1-K22A and rBmTX4-P1-Y31A at a concentration of 1 µM on hKv1.2 channels. Each channel was tested at least three times. The results are shown as the mean ± SE. (**G**) The inhibition rates of hKv1.2 channel currents by 1 μM rBmTX4-P1 and its mutants. (**H**) The secondary structure of rBmTX4-P1, in which the two functional amino acids are highlighted. (**I**) The secondary structure of BmKcug2-P1, in which the two functional amino acids are highlighted.

To further identify the functional sites of BmTX4-P1 interacting with the potassium channel, a series of mutant peptides of BmTX4-P1 were prepared to test their inhibitory activity on potassium channels. Through sequence alignment, we found that the sequence of BmTX4-P1 is highly similar to that of BmKcug2 and its truncated analogs. In addition, BmKcug2 and BmKcug2-P1 all show the highest affinity for the hKv1.2 channel [22]. Lys^27^ and Tyr^36^ of BmKcug2 and its truncated analog BmKcug2-P1, which act as the classical “functional dyad” of potassium channel-inhibiting scorpion toxins [22,23,24], are crucial for the high affinity to the hKv1.2 channel. As Lys^22^ and Tyr^31^ of BmTX4-P1 correspond to Lys^27^ and Tyr^36^ of BmKcug2 and its truncated analog BmKcug2-P1, we prepared two mutant peptides of BmTX4-P1 (BmTX4-P1-K22A and BmTX4-P1-Y31A) by recombinant expression (Figure 6A,D). Then, they were confirmed by mass spectrometry, and the results corresponded to their calculated value, indicating that rBmTX4-P1-K22A and rBmTX4-P1-Y31A were successfully produced (Figure 6B,E). By evaluating the blocking activities of rBmTX4-P1-K22A and rBmTX4-P1-Y31A acting on the hKv1.2 channel, two mutant peptides of BmTX4-P1 exhibited a dramatic drop in affinity for the hKv1.2 channel (Figure 6C,F). A total of 1 μM rBmTX4-P1-K22A and rBmTX4-P1-Y31A only inhibited 3.2 ± 1.1% and 4.3 ± 0.6% of potassium currents mediated by the hKv1.2 channel, respectively (Figure 6C,F,G). These results suggested that the Lys^22^ and Tyr^31^ of BmTX4-P1, as the classical “functional dyad” of potassium channel-inhibiting scorpion toxins, were indispensable for the high affinity to the hKv1.2 channel, as expected. This result further indicated that BmTX4-P1 exhibits a similar sequence and functional site to BmKcug2-P1 (Figure 6H,I).

## 3. Discussion

Thermally processed *Buthus martensii* Karsch scorpion is an important animal medicinal material commonly used in traditional Chinese medicine. The source and processing of scorpion medicinal material are commonly as follows: the live *Buthus martensii* Karsch scorpions were captured from late spring to early autumn. After removing sediment, they are placed in boiling water or boiling saltwater until they become stiff all over the body. Then, they were removed from boiling water or boiling saltwater and dried in a shaded and ventilated place [1]; by this method, the obtained dry body is the traditional Chinese medicinal material named “Quanxie”. It has been used to treat a variety of diseases for more than one thousand years. However, until now, little was known about the active substances in this material, which has seriously limited the modern medicinal use of traditional Chinese scorpion medicine materials. Based on the Chinese saying “combat poison with poison”, the venom gland in scorpion medicinal material is an effective medicinal part. Several years ago, a peptidomic study on the venom gland of traditional Chinese scorpion medicinal materials indicated that a large number of degraded peptides from potassium channel-interacting scorpion toxins still occurred in the venom glands of traditional Chinese scorpion medicinal materials [13]. As only a few studies have been carried out on the degraded peptides in the venom gland of traditional Chinese scorpion medicinal materials [13,22,25], much research is still needed to determine the peptide components and their pharmacological properties in traditional Chinese scorpion medicinal materials.

An increasing number of potassium channels have been found to be closely related to the pathogenesis of human diseases [16,17,21,26], such as the Kv1.2 and Kv1.3 channels; these channels were found to be closely related to epilepsy and may become new targets for epilepsy treatment [17,21]. The discovery of peptides acting on potassium channels from traditional Chinese scorpion medicinal material is of great significance for unveiling the effective components of this medicinal material. In addition, the peptide blockers found in this medicinal material for potassium channels could serve as channel-identifying pharmacological tools or channelopathy-treating candidate drugs. Interestingly, previous studies have reported that hKv1.2 and hKv1.3 channels are implicated in gastrointestinal diseases [27,28,29,30,31], and the inhibitor cystine knot (ICK) peptide linaclotide can treat the irritable bowel syndrome with constipation by targeting the adenylate cyclase 2C receptor in the gastrointestinal tract [32]. In addition, ICK peptides have been reported to be resistant to proteolytic enzymes [33,34]. So, whether these thermostable BmTX4-P1, BmKcug2, and BmKcug2-P1 peptides are resistant to the digestive enzymes and can treat gastrointestinal diseases by inhibiting Kv1.2 and Kv1.3 channels would be an interesting work in the future [13,22].

In this study, a new degraded peptide, BmTX4-P1, was identified in traditional Chinese scorpion medicinal materials by proteomics. Then, sBmTX4-P1 was prepared by chemical synthesis, and pharmacological experiments revealed that sBmTX4-P1 was a highly potent inhibitor of the hKv1.2 channel and hKv1.3 channel with IC_50_ values of 74.7 ± 7.0 nM and 123.9 ± 26.7 nM, respectively. To further investigate the functional residues of BmTX4-P1, the recombinant BmTX4-P1 peptide, and its mutant peptides were prepared by recombinant expression. However, probably because the N-terminus of degraded BmTX4-P1 starts with lysine (K) and is close to the CS α/β motif, it is difficult to obtain rBmTX4-P1 by recombinant expression, as recombinant enterokinase cannot cleave the fusion protein at the only correct site to obtain rBmTX4-P1. We found that there is also a secondary enterokinase cleavage site in the fusion protein, as we have described previously [35]. The correct cleavage site of enterokinase is after the amino acid sequence “DDDDK”, but it incorrectly cleaves after the secondary cleaved site with the amino acid sequence “ER”, which is located in the region of the 15 aa S-Tag “KETAAAKFERQHMDS” (Figure 4B,C). Thus, a new expression vector was constructed by deleting the base sequence “GAACGC” of the expression vector, which resulted in the deletion of the wrong recognition site “ER” of enterokinase in the fusion protein (Figure 4C). Finally, rBmTX4-P1 was successfully obtained by recombinant expression with the new expression vector. The pharmacological experiments of rBmTX4-P1 showed that we prepared the functional peptide rBmTX4-P1 and that rBmTX4-P1 inhibited hKv1.2 and hKv1.3 channels with IC_50_ values of 45.7 ± 9.2 nM and 246.8 ± 47.9 nM, respectively (Figure 5). The mass spectrometry analysis indicated that the same molecular weights of sBmTX4-P1 and rBmTX4-P1 were the same, while their IC_50_ values on hKv1.2 and hKv1.3 channels showed minor differences, likely caused by the impurities in the rBmTX4-P1 peptide, whose mass spectrum appeared together with some mass spectra impurities. The pharmacological activities of sBmTX4-P1 and rBmTX4-P1 all indicate that BmTX4-P1 is more selective for the hKv1.2 channel. Therefore, we further investigated the functional sites of BmTX4-P1 involved in the interaction with the hKv1.2 channel. BmTX4-P1-K22A and BmTX4-P1-Y31A were prepared by recombinant expression, and the pharmacological activities of BmTX4-P1-K22A and BmTX4-P1-Y31A indicated that two critical amino acids (i.e., Lys^22^ and Tyr^31^) of BmTX4-P1 determined the activity on the hKv1.2 channel (Figure 6). Our work showed that the degradation peptide BmTX4-P1 was identified by MS/MS spectra analysis from traditional Chinese scorpion medicine materials, but the corresponding venom-derived peptide toxin BmTX4 was not found in traditional Chinese scorpion medicine materials. So, in this work, our pharmacological studies were focused on the degradation peptide BmTX4-P1. In addition, because the degradation peptide BmTX4-P1 came from the venom-derived peptide toxin BmTX4, and BmTX4 peptide possesses the two critical amino acids that have been identified from BmTX4-P1 (K22 and Y31), our present work also suggested that BmTX4 may also be a potent potassium channel inhibitor. In general, this research not only identified a new degraded peptide, BmTX4-P1, from traditional Chinese scorpion medicinal material that showed high inhibitory activity against hKv1.2 and hKv1.3 channels but also successfully used two methods (chemical synthesis and recombinant expression) to obtain sBmTX4-P1 and rBmTX4-P1 peptides; thus, the research laid the foundation for preparing degraded peptides from processed *Buthus martensii* Karsch scorpions. Overall, this research expanded our knowledge of the degraded peptides from traditional Chinese scorpion medicinal material and will be helpful for studying the medicinal function of degraded peptides from processed *Buthus martensii* Karsch scorpions.

## 4. Materials and Methods

### 4.1. Scorpion Toxin Extraction and LC–MS/MS Analysis

We purchased traditional Chinese scorpion medicinal materials from a pharmacy for traditional Chinese medicine. The extract of venomous telsons was prepared according to previous procedures [13]. Finally, LC–MS/MS analysis was applied to identify peptide sequences in extracts of venomous telsons of traditional Chinese scorpion medicinal materials after reduction and alkylation, which was described in detail in previous work [13].

### 4.2. Chemical Synthesis of sBmTX-P1

sBmTX-P1 was chemically synthesized in its oxidative form by Smartox Biotechnology (Saint Egrève, France). To ensure the correct pattern of disulfide bond formation, peptides were synthesized chemically with cysteine protected by orthogonal groups. The bridges were formed one by one. Finally, the number of bridges was controlled by mass spectrometry.

### 4.3. Source of Potassium Channel Plasmids

The source of potassium channel plasmids was described in detail in previous articles [22]. The cDNA encoding hKv1.1 channel, hKv1.3 channel, and rKv4.3 channel were subcloned into the vector of pCDNA3.1(+). The cDNA encoding hKv1.2 channel, hKv1.6 channel, hKv3.2 channel, hERG channel, and mKv4.1 channel were subcloned into the vector of pIRES2-EGFP. The cDNA encoding rKv4.2 was subcloned into the vector of pEGFP-N1.

### 4.4. Cell Culture and Expression of Potassium Channels

DMEM (Gibco, Pittsburgh, PA, USA) with 10% fetal bovine serum and 1% penicillin/streptomycin was applied to culture HEK293T cells in an incubator with 5% CO_2_ at 37 °C. According to the manufacturer’s instructions, ExFect Transfection Reagent (Vazyme, Nanjing, China) was used to co-transfect the plasmids of pEGFP-N1 and potassium channels into HEK293T cells. Fluorescence-positive cells were selected for electrophysiological experiments after transfection for 12 h to 24 h.

### 4.5. Electrophysiological Recordings

Whole-cell channel currents were measured and recorded by an EPC-10 patch-clamp amplifier controlled by PatchMaster (HEKA Elektronik, Lambrecht, Germany). The internal patch pipette solution and bath solution were prepared according to the protocol in previously published references [13]. Bath solution with 0.01% BSA (Gibco, Pittsburgh, PA, USA) was used to dissolve peptides for electrophysiological testing. The external recording bath solution was exchanged by the MPS-2 multichannel microperfusion system (INBIO Inc., Wuhan, China). The currents of different potassium channels were generated and recorded following the respective protocols according to previously published references [13]. The results of the electrophysiological experiment are presented as the mean ± SE, and more than three cells from each sample were examined (n ≥ 3).

### 4.6. Construction of the rBmTX4-P1 Peptide Expression Vector

The cDNA of BmTX4-P1 was amplified by overlapping PCR. The pET-32a (+) expression vector and the cDNA of BmTX4-P1 were digested by endonuclease of Kpn I and Xho I. The cDNA of BmTX4-P1 would be inserted into the pET-32a (+) expression vector by T4 DNA ligase (Takara, Japan). A QuikChange Site-Directed Mutagenesis Kit (Stratagene, CA, USA) was applied to obtain the mutant plasmids based on the recombinant plasmids of BmTX4-P1. All recombinant plasmids were transformed into *E. coli* Rosetta (DE3) for expression.

### 4.7. Expression and Purification of Peptides

The prokaryotic expression system was applied to prepare the recombinant peptides. The *E. coli* Rosetta (DE3) cells into which recombinant plasmids were transformed were cultured in LB medium with 50 μg/mL ampicillin at 37 °C. When the DE3 cells reached their logarithmic growth phase, 1 mM isopropyl β-D-thiogalactopyranoside (IPTG) was added to induce the expression of fusion protein at 25 °C for 12 h. Then, the bacterial cells were collected by centrifugation and resuspended in chilled 20 mM imidazole buffer (pH = 7.9). An ultrasonic crusher was applied to crack the DE3 cells, and then the His-Tag affinity chromatography was applied to purify the fusion protein. After the fusion proteins were concentrated by ultrafiltration, they were digested with enterokinase (Sangon Biotech, Shanghai, China) at 25 °C for 12–16 h. High-performance liquid chromatography was applied to isolate the target peptides using a linear gradient from 5% to 95% acetonitrile (ACN, Millipore, Burlington, MA, USA) and 95% to 5% Milli-Q water with 0.1% trifluoroacetic acid (TFA, Sigma, Marlborough, MA, USA) over 60 min on a C18 column (10 × 250 mm, 5 μm; Elite-HPLC, Dalian, China), and the absorbance was detected at 230 nm. The matrix-assisted laser desorption ionization time-of-flight mass spectrometry (MALDI-TOF-MS) was applied to identify the molecular weights of purified peptides. Finally, we subpackaged the confirmed peptides and stored them at −80 °C.

### 4.8. Data Analysis

The software of Clampfit (Molecular Devices, Sunnyvale, CA, USA) and Sigmaplot (IBM SPSS, Chicago, IL, USA) were applied to analyze the electrophysiological data. In order to obtain IC_50_ values, the concentrations of peptides and currents of potassium channels were used to fit the modified Hill equation for dose–response relationships: I_peptide_/I_control_ = 1/[1 + C_peptide_/IC_50_], in which I_peptide_ and I_control_ represent the peak tail current of potassium channels in the presence and absence of peptide, C_peptide_ represents the concentration of peptides and IC_50_ represents the half-maximum inhibition concentration. The results are presented as the mean ± SE.

## Data Availability

All data supporting the results can be found within the manuscript.
